# The association between black stain and lower risk of dental caries in children: a systematic review and meta-analysis

**DOI:** 10.1186/s42506-022-00107-3

**Published:** 2022-07-30

**Authors:** Haneen Raafat Fathi Mousa, Mohamed Zayed Radwan, Ghada Ossama Mohamed Wassif, Mariem Osama Wassel

**Affiliations:** 1 Pediatric Dentistry and Dental Public Health Department, Faculty of Dentistry, Ain Shams University, Organization of African Union Street, Post NO.: 11566, Abbasia, Cairo, Egypt; 2 Department of Community, Environmental, and Occupational Medicine, Faculty of Medicine, Ain Shams University, Abbasia, Cairo, Egypt

**Keywords:** Dental caries, black stain, extrinsic stain, discolored plaque, primary dentition

## Abstract

**Background:**

Previous literature shows that children with dental black stain might be less susceptible to dental caries. The aim of this study was to systematically review the available literature to determine whether black stain presence could influence the prevalence or severity of dental caries in primary dentition.

**Methods:**

A systematic search of PubMed, Web of Science, Scopus, Google Scholar, OpenGrey, and Egyptian Universities Libraries Consortium was conducted up to December 2020. Quality assessment was done using a modified version of Down’s and Black checklist. Meta-analyses were performed to assess the association between dental black stain and: (i) Likelihood of developing dental caries/being caries-free (ii) Number of teeth affected by dental caries (iii) Number of tooth surfaces affected.

**Results:**

The database search yielded 2164 results, 14 of which matched the eligibility criteria. The meta-analysis showed that the likelihood of developing caries (Fixed effect model: OR [95% CI]: 0.67 [0.54; 0.82]; I^2^=37%; τ^2^=0.05), number of teeth affected (Random effects model: MD [95% CI]: –0.98 [–1.54; -0.42]; I^2^=79%; τ^2^ =0.44), and number of surfaces affected (Random-effects model: MD [95% CI]: –2.34 [–4.23; -0.44]; I^2^=85%; τ^2^ =2.93), were all lower in children with black stain.

**Conclusions:**

It is suggested that dental black stain is associated with lower dental caries experience in children with primary dentition. However, it is questionable whether black stain has a protective effect against dental caries, or whether children at low risk of dental caries are more likely to develop BS because their oral microbiome favors BS-forming organisms.

**Supplementary Information:**

The online version contains supplementary material available at 10.1186/s42506-022-00107-3.

## Introduction

Dental discoloration constitutes a major esthetic problem, particularly when anterior teeth are significantly affected [1]. Such condition could have long-lasting serious negative consequences in younger individuals, in particular, since compromised dental esthetics during childhood or adolescence could affect the psychosocial development of children, and interfere with their interaction with peers [2].

Black stain (BS) is one of the common types of extrinsic discolorations that affect pediatric patients [3]. Clinically, BS has a very characteristic appearance that appears as black deposits of incompletely fused black dots or lines on the enamel surface and is more commonly encountered on the cervical one third of affected teeth but can appear elsewhere. It affects both adults and children but is more commonly seen in children [3]. Despite not being associated with any functional impairment, it has constantly been regarded as a source of esthetic concern for children and their families [4], all the more because of its high recurrence tendency [5].

In the early literature, bacterial species such as *Prevotella nigrescens, Prevotella melaninogenica, Prevotella intermedia, Porphyromonas gingivalis,* and *Actinomyces spp* were frequently isolated and cultured from black stain deposits [6, 7]. PCR studies confirmed the presence of *Actinomyces* spp, [8–10] although some showed that the counts were not significantly different from healthy children. However, as of yet, the causative organism of BS remains speculative. As for composition, it is widely believed that the BS deposits are made of an iron compound. It was proposed that this compound could be ferric sulfide, formed as a result of the reaction between hydrogen sulfide—which is a bacterial product—and the iron in the saliva or crevicular fluid [11].

Many risk factors have been associated with BS development in children. It was suggested that an iron-rich diet, iron supplements, or iron-containing medications could be possible reasons for BS [12–14]. The consumption of chromogenic food materials was also thought to be linked to developing BS [15]. Nevertheless, it is not yet understood why this stain can affect some children who are not exposed to such factors.

Black Stain is also believed to be associated with reduced dental caries experience of children [4, 15]. The assumption that BS could be a sign of immunity against dental caries was first made as early as the beginning of the 20^th^ century [3]. Some authors even suggested that accepting BS to some extent, if it is proven to be more beneficial biologically, should be more preferable than the conventional ideal image of “clean white teeth” [16]. However, it’s yet to be understood how does BS presence affects the dental decay process.

Thus, the aim of this study was to answer the the research question “Is the presence of black stain on primary teeth associated with the severity or extent of dental decay compared to teeth free of such stains?”. This was done through testing whether there’s a difference between children with BS and children free of BS in: (i) Likelihood of developing dental caries/being caries-free (ii) Number of teeth affected by dental caries (iii) Number of tooth surfaces affected by dental caries.

## Methods

### Protocol registry

The study was reported according to the PRISMA checklist 2009 [17], and a study protocol was registered at PROSPERO-international prospective register of systematic reviews, registration ID: CRD42020111.

### Search strategy

A systemic search was conducted in the online databases: PubMed (PM), Web of Science (WoS), Scopus, Google Scholar (GS), OpenGrey (OG), and the Egyptian Universities Libraries Consortium (EULC). To evaluate the search strategy, preliminary searches were perfomed starting the 15^th^ of January 2020, and the search was last run for all databases on the 31^st^ of December 2020.

Reference lists of all eligible records were manually searched to identify any additional records meeting the prespecified eligibility criteria of this review. Only studies reported in the English language were assessed for eligibility, but no time restrictions were applied in all databases.

### Search terms

The search terms used for black stain included: Black stain, BS, black pigmentation, black discoloration, black tartar, while search terms for dental caries included: dental caries, carious teeth, carious lesions, cavities, dental decay, dmfs, dmft, defs, deft, dfs, dft. Target population was defined using the search terms: children, pediatric, students, pupils, preschool, primary dentition, deciduous teeth. The detailed search strategy for each database is available in (Table S1 – supplemental material).

### Eligibility criteria

Publications comparing dental caries prevalence or severity in the primary dentition between children with and without black stain were included. Prevalence of dental caries was assessed through: (i) presence of dental caries, (ii) number of teeth affected. While the severity of dental caries was assessed through the number of surfaces affected. Inclusion and exclusion criteria are listed in Table 1.

**Table 1:** Inclusion and exclusion criteria of the studies

Inclusion Criteria	Exclusion Criteria
1. Observation studies (cross-sectional, case-control, retrospective cohort, or prospective cohort). 2. Studies assessing primary teeth. 3. Studies reported in the English language. 4. Studied having the following **PECOs**: a. Population: children with primary or mixed dentition. b. Exposure: black stains of any amount or extent related to primary teeth. c. Comparators: children without dental black stains. d. Outcomes: Occurrence of dental caries, number of teeth affected, number of surfaces affected.	1. Studies where caries parameters are not described in terms of the type of dentition. 2. Self-reported dental caries or black stains. 3. Studies that were conducted on medically compromised or institutionalized children. 4. Case-reports and case-series. 5. Editorials, and commentaries. 6. Secondary literature sources (such as books or reviews)

### Screening and study-selection

All records were imported to EndNote Web and checked for duplicates. After duplicate removal, records were screened through a two-phase process. Title/abstract screening was conducted by MW and HM independently, while full-text screening was done by MR and HM independently. Disagreements were all resolved in the end by the most senior investigator MW.

### Data extraction

Data extracted from eligible records were tabulated in predesigned standard forms. The extracted data included: author names, date of publication, study type, number of participants in each study, age of participants, country, setting, prevalence of BS in the sample population (for cross-sectional studies), and effect size measures for children with and without BS. Data were extracted by two independent investigators: GW and HM.

### Quality assessment

Methodological quality assessment was done using a 15-item modified version of Down’s and Black checklist [18] that was adapted by Zadro et al. (2017) [19] The scoring system was yes = (1), and no or unable to determine = (0), for all items except item 4, where yes = (2), partially = (1), and no = (0). The maximum attainable score for any record was 16. As suggested by previous studies [20, 21] a cut-off point of 50% for methodological quality was adopted. Studies scoring 8 out of 16 or less were excluded from the meta-analyses. The quality assessment of all records was done by two investigators: GW and HM independently, and disagreements were resolved by MW.

### Statistical analysis

All statistical analyses and diagrams were performed through RStudio Version 1.1.463 – © 2009-2018 RStudio, Inc. (open-source license), using the statistical packages “meta” and “metaphor.” Data regarding effect estimates and dispersion estimates were extracted from the studies, organized into Excel spreadsheets, and imported into RStudio.

Meta-analyses were performed using the mean difference for continuous outcomes, and odd’s ratio for binary outcomes. Heterogeneity was assessed using Higgin’s & Thompson’s I^2^ and Tau-squared (τ^2^). Estimation of between studies variance (τ^2^) was done through the DerSimonian-Laird approach. P-value of the significance of I^2^ was set at p-value ≤ 0.05, random effects model was used when significant heterogeneity was found. Publication bias was tested using contour-enhanced funnel plots for each study outcome.

### Outcome definition

Children with BS were designated as the experimental/intervention group, whereas children with no BS were designated as the control group. (i) Likelihood of caries development was a dichotomous outcome (present/absent). (ii) Number of affected teeth was estimated based on tooth-level estimates (such as dmft, deft, or dft). (iii) dental caries severity was calculated based on surface-level estimates (such as dmfs, defs, or dfs). Separate meta-analyses were also done according to study type. Whenever multiple outcome measures were reported for the same outcome in a study, the outcome measure with a higher number of components was chosen to be included in the meta-analysis (for example dmft would be chosen over dft, if both were reported in the same study).

## Results

### Search results

**Fig. 1:**
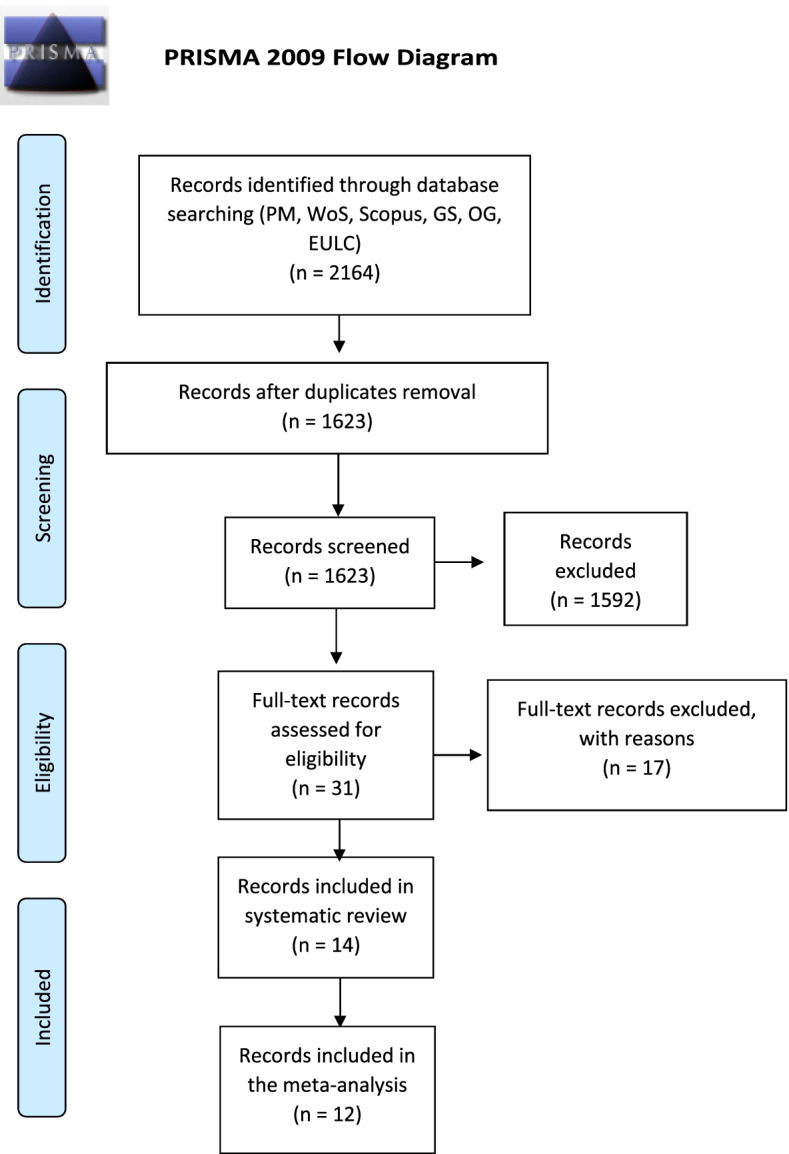
PRISMA flowchart of methodology used to obtain eligible studies

The database search yielded 2164 records in total (PM=238, WoS=289, Scopus= 1104, GS=530, OG= 0, EULC=3; last accessed 31^st^ of December 2020). After the removal of duplicated records, 1623 records remained to be screened for eligibility. After title/abstract screening, 1,592 records were excluded, while 31 were considered for full-text assessment. Subsequently, 17 full-text records were excluded (Table S2 – Supplemental material), while 14 records were included in the systematic review. PRISMA Flow diagram of the study selection process is shown in (Fig. 1).

Data extracted from the included studies are listed in Table 2. Out of the 14 studies, eight studies were cross-sectional, six were case-control, and none were cohort studies. Seven studies reported presence of dental caries, eleven studies reported tooth-level outcome measures, and four studies reported surface-level outcome measures.

**Table 2:** Characteristics of the included studies

	Author/date	Study design	Count-ry	Setting	Age	Sample size	Diagnosis method and criteria	Prevalence of BS	Prevalence of caries (BS)	Prevalence of caries (no BS)
1	{Akyuz, 2015} [22]	Cross-sectional	Turkey	One university clinic	5 -13	325	Clinical exam Caries (dft) BS (yes/no)	18.5%	dft = 3.67 (3.25)^a^ Presence of caries= 86.67%	dft = 4.29 (3.48)^a^ Presence of caries= 92.08%
2	{Garan, 2012} [23]	Case- control	Turkey	One university clinic	7-12	38	Clinical exam Caries (dft) BS (yes/no)	N/A	dft= 1.21 (2.04)^a^	dft= 3.60 (3.60)^a^
3	{Boka, 2013} [24]	Cross-sectional	Greece	20 kinder-gartens	3- 5.5	804	Clinical exam Caries (dmfs) BS (yes/no)	2.4%	dmfs = 0.38 (0.9)^a^	dmfs = 1.19 (3.9)^a^
4	{Chen, 2014} [15]	Cross-sectional	China	12 kinder-gartens	4.55	1,397	Clinical exam Caries (dmft and dmfs) BS (yes/no)	9.9 %	dmft= 1.91 (3.08)^a^ dmfs= 4.22 (7.84)^a^ Presence of caries= 46.4 %	dmft= 2.97 (3.91)^a^ dmfs= 6.69 (10.15)^a^ Presence of caries= 59.1 %
5	{Elelmi, 2020} [25]	Cross-sectional	Tunisia	21 kindergartens	3-5	393	Clinical exam Caries (dmft) and ECC (yes/no) BS (yes/no)	6.1%	dmft= 0.83 (SD not reported) ECC=33.3%	dmft= 1.46 (SD not reported) ECC=50.9%
6	{França-Pinto, 2012} [26]	Cross-sectional	Brazil	Homes	5	1,120	Clinical exam Caries (dmfs) BS (yes/no)	3.5%	dmfs= 3.3 (6.7)^a^ Presence of caries= 41.03%	dmfs= 4.1 (7.4)a Presence of caries= 48.66%
7	{Garcia Martin, 2013} [12]	Cross-sectional	Spain	One health center	6	3,272	Clinical exam Caries (dmft) BS (yes/no)	3.1%	dmft= 0.35 (1.123)^a^	dmft= 0.65 (1.852)^a^
8	{Heinrich-Weltzien, 2014} [10]	Case- control	Germany	A kinder-garten and an elementary school	7.9 ± 1.3	93	Clinical exam Caries (dmft) BS (yes/no)	N/A (1.5% in an earlier study in 2011 from which the participants were chosen)	dmft=1.6 (2.1)^a^ Presence of caries= 48.9%	dmft= 3.0 (3.2)^a^ Presence of caries= 67.4%
9	{Hwang, 2020} [27]	Case- control	Korea	-	4-11	10	Clinical exam Caries (dmft) BS (Bs scale 0,1,2)	N/A	dmft= 3.8 (SD not reported)	dmft= 1.75 (SD not reported)
10	{Koch, 2001} [28]	Cross-sectional	Italy	Elementary schools	6-12	1086	Clinical exam Caries (dmft) BS (yes/no)	6.17%	dmft= 1.87 (2.47)^a^	dmft= 2.39 (2.62)^a^
11	{Muthu, 2019} [29]	Cross-sectional	India	150 Anganwadi centers	0-3	1,486	Clinical exam ECC (yes/no) BS (yes/no)	6.2%	ECC=43.48%	ECC=40.6%
12	{Mutsaddi, 2018} [30]	Case- control	India	Schools	7 -11	60	Clinical exam Caries (dft and dfs) BS (yes/no)	N/A	dft= 0.80 (1.32)^a^ dfs= 1.30 (2.45)^a^	dft= 4.33 (3.57)^a^ dfs= 7.73 (7.19)^a^
13	{Sharaf, 2017} [31]	Case- control	Egypt	One university clinic	3-12	80	Clinical exam Caries (dmft/deft) BS (yes/no)	N/A	dmft/deft= 4 (3 - 6)^b^	dmft/deft= 2 (0 - 4)^b^
14	{Tripodi, 2016} [32]	Case-control	Italy	One university clinic	9.82 ±4.43	189	Clinical exam Caries (dmft) BS (yes/no)	(1.8% of the whole study population)	dmft= 0.12 (3.15)^a^ Presence of caries= 26,08%	dmft= 0.1 (3.07)^a^ Presence of caries= 44,12%

*^a^Mean (SD), ^b^Median (IQR)

### Quality assessment

Modified Down’s and Black checklist scores ranged from 5 to 15 (mean score=10.9) out of a maximum attainable score of 16. (Chen, 2014) [15] had the highest score (15/16), whereas (Hwang, 2020) [27] had the lowest score (5/16), however it should be noted that evaluating the clinical relation between dental caries and black stains (upon which quality assessment was based) was not the main objective of this study. Consequently, (Hwang, 2020) was excluded from the meta-analyses on basis of quality. Individual study scores are shown in Table 3.

**Table 3:** Modified downs and black scores

	Author/date	Total (16)		Author/date	Total (16)
1	{Akyuz, 2015} [22]	11/16	8	{Heinrich-Weltzien, 2014} [10]	9/16
2	{Garan, 2012} [23]	10/16	9	{Hwang, 2020} [27]	5/16
3	{Boka, 2013} [24]	14/16	10	{Koch, 2001} [28]	10/16
4	{Chen, 2014} [15]	15/16	11	{Muthu, 2019} [29]	9/16
5	{Elelmi, 2020} [25]	11/16	12	{Mutsaddi, 2018} [30]	9/16
6	{França-Pinto, 2012} [26]	13/16	13	{Sharaf, 2017} [31]	13/16
7	{Garcia Martin, 2013} [12]	12/16	14	{Tripodi, 2016} [32]	11/16

### Meta-analyses

**Fig. 2:**
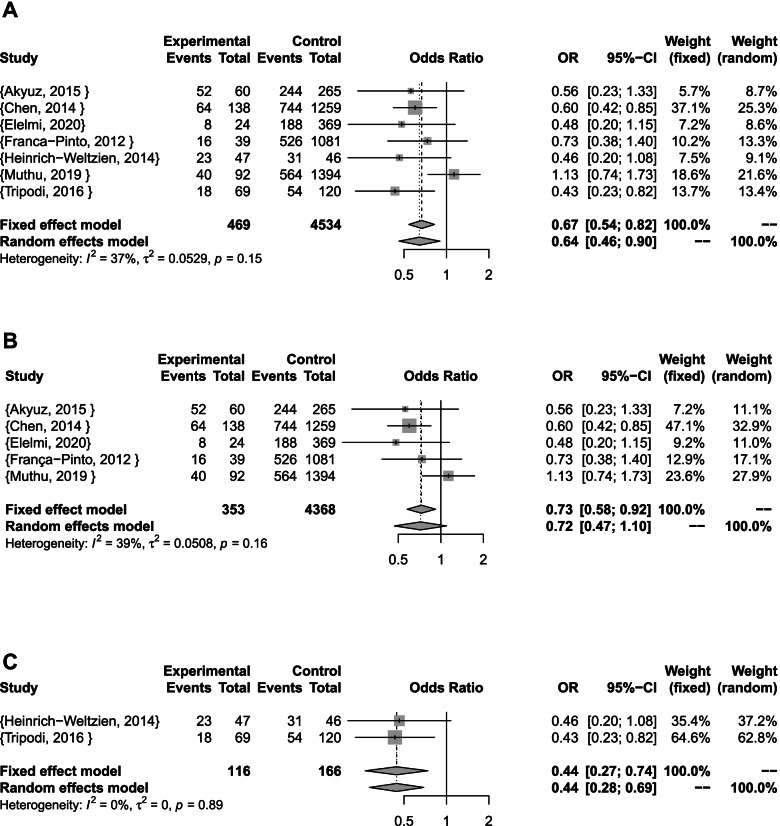
Caries development meta-analyses **A** Combined **B** Cross-sectional studies **C** case-control studies

The pooled result of caries development in all types of studies showed a statistically significant associaciation with lower odds of developing dental caries (Fixed effect model: OR [95% CI]: 0.67 [0.54; 0.82]; I^2^ = 37%; p = 0.15; τ^2^ = 0.05) The results were also statistically significant for cross-sectional studies (Fixed effect model: OR [95% CI]: 0.73 [0.58; 0.92]; I^2^ = 39%; p = 0.16; τ^2^ = 0.05) , and case-control studies (Fixed effect model: OR [95% CI]: 0.44 [0.27; 0.74]; I^2^ = 0%; p = 0.89; τ^2^ = 0) (Fig. 2).

**Fig. 3:**
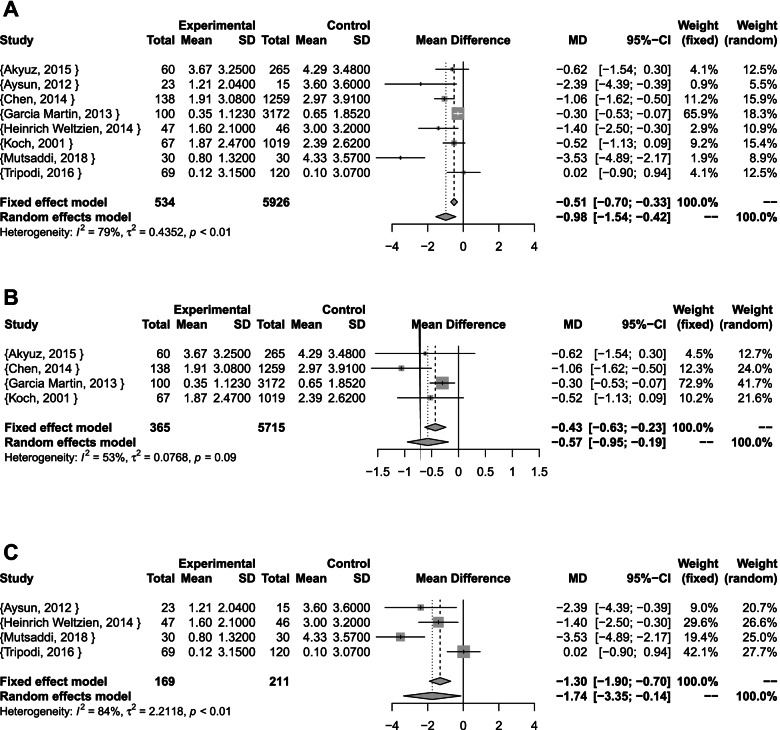
Tooth-level meta-analyses **A** Combined **B** Cross-sectional studies **C** case-control studies

The pooled results of tooth-level estimates for all studies showed a statistically significant mean difference of almost one less primary tooth with caries experience in case of BS (Random effects model: MD [95% CI]: –0.98 [–1.54; -0.42]; I^2^ = 79%; p < 0.01; τ^2^ = 0.44). The results were also statistically significant for cross-sectional studies (Fixed effect model: MD [95% CI]: –0.43 [–0.63; -0.23]; I^2^ = 53%; p = 0.09; τ^2^ = 0.08), and case-control studies (Random effects model: MD [95% CI]: –1.74 [–3.35; -0.14]; I^2^ = 84%; p < 0.01; τ^2^ = 2.21) (Fig. 3).

Three studies were not included tooth-level meta-analyses. (Elelmi, 2020) was excluded since SD was not reported, but was included in caries development meta-analysis. (Hwang, 2020) was excluded due to methodological quality. (Sharaf, 2017) was not included because of difficulty of interpreting the data since effect estimates were only reported using the median scores and Interquartile range (IQR).

**Fig. 4:**
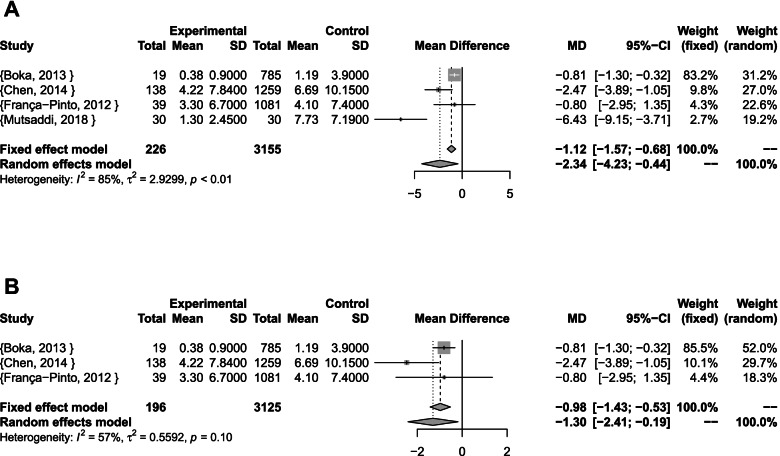
Surface-level meta-analyses **A** Combined **B** Cross-sectional studies

The pooled results of surface-level estimates for all studies showed a statistically significant mean difference of at least two less primary teeth surfaces with caries experience in case of BS (Random-effects model: MD [95% CI]: –2.34 [–4.23; -0.44]; I^2^ = 85%; p < 0.01; τ^2^ = 2.93). The results were also statistically significant for cross-sectional studies (Fixed-effect model: MD [95% CI]: –0.98 [–1.43; -0.53]; I^2^ = 57%; p = 0.1; τ^2^ = 0.56), (Fig. 4).

### Publication bias analysis

**Fig. 5:**
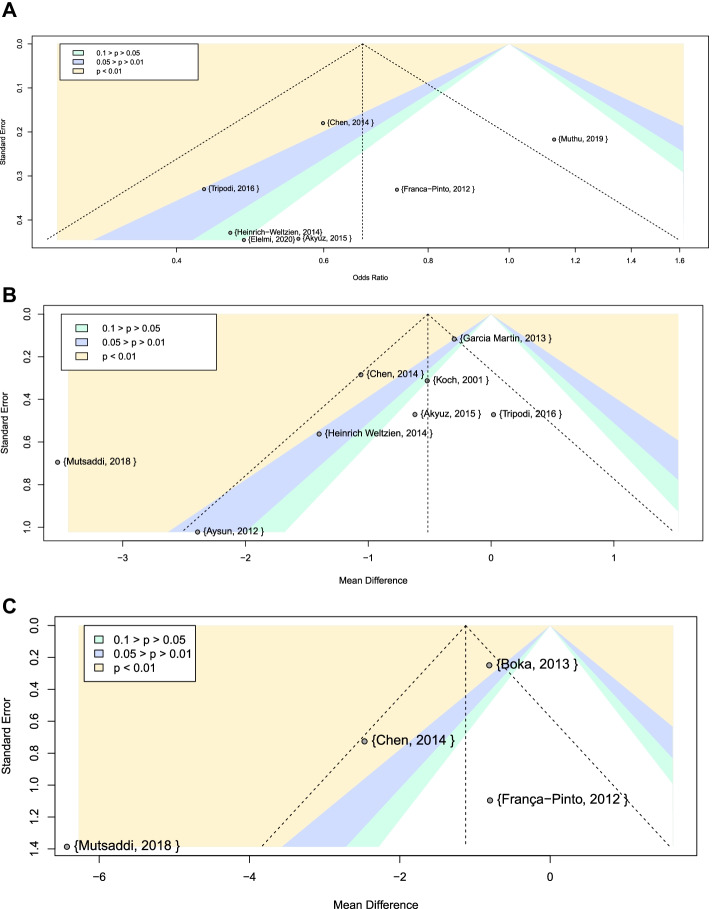
Contour-enhanced funnel plot **A** Caries development **B** Tooth-level **C** Surface level

Visual assessment of the funnel plots (Fig. 5) shows asymmetry, as few studies occupy the lower right part of the plots, and the smaller studies with higher SE values tended to coincide with areas of statistical significance, which could indicate the possibility of publication bias. However, a larger number of studies is needed to confirm this conclusion.

## Discussion

Throughout literature, many attempts were made to investigate the relationship between BS and dental caries, but as of yet, little is known about the exact mechanism through which BS presumably protects human teeth from decay. Slots (1974 ) [6] suggested two postulates: (1) Actinomycetes—supposedly causing the BS—could be responsible for the low caries frequency, or (2) the presence of the *Actinomycetes* and BS is only secondary to an unknown independent caries-inhibiting factor present in the oral cavity, and thus lower counts of *Streptococci* are observed in individuals with BS. Reid et al. (1977 ) [11] attributed the lower caries frequency to the difference in microbiome composition and metabolism. In addition to this, it was assumed that the higher concentrations of calcium and phosphorus—compared to normal plaque—decreased the solubility of tooth enamel and increased the buffering capacity, ultimately preventing dental caries.

High iron levels were also thought to contribute to the caries-protective effect. Iron has been frequently reported to decrease the cariogenicity of sugars [33–36]. Other salivary factors such as higher buffering capacity [23], or higher pH [3], were also proposed as potential pathways through which BS could reduce dental caries activity.

In this study, the quantitative synthesis of the available evidence suggests that children with BS deposits on their primary dentition are less likely to develop dental decay, and experience less decay per surface and per tooth than their peers without this type of staining. This was demonstrated in cross-sectional, case-control, as well as combined syntheses of different study types, which suggests that the presence of BS might actually interfere somehow with the dental caries process.

Only observational studies were included in this review due to the nature of conditions being studied. Eight cross-sectional studies, and six case-control studies were identified; however, no longitudinal studies were found. It is quite well-known that longitudinal studies are superior to non-longitudinal observational studies, because of the ambiguity of the temporal order of exposure and outcome [37].

In our case, point estimates of dental decay reflect the total caries experience of the child before as well as after encountering BS, and thus, it is not clear how much the child’s caries experience is actually influenced by the presence of BS. Furthermore, longitudinal studies could have also provided valuable insights into the factors associated with aggravation, or spontaneous resolve of BS in pediatric patients, since BS has frequently been reported to resolve spontaneously with age by some authors [32].

Five of the studies included in this review were single-center studies [12, 22, 23, 31, 32], which could compromise the external validity of these studies [38]. It is also noteworthy that six studies in this systematic review were conducted in healthcare facilities [12, 22, 23, 29, 31, 32], four of which were dental university clinics [22, 23, 31, 32]. Recruiting participants from hospital settings could be regarded as a source of selection bias [39]. Children present in dental care facilities are likely to exhibit higher levels of dental caries. Caries-free children might also present at dental care facilities, if they have esthetic problems, such as those caused by BS, which means that the difference in dental caries experience between those with and without BS might not be generalizable on the general population.

Clinical examination was used by all the studies to assess the presence or absence of BS. Consequently, patients meeting the diagnostic criteria were considered “cases,” whereas those who do not were considered “controls” or “comparators.” However, since the tendency of BS to recur is a well-documented phenomenon [5], children who might have received recent treatment for BS, could have been easily misplaced into control groups, as none of the studies reported inquiring about the past history of BS presence. It should be noted that a biased detection of the participants’ exposure status constitutes as misclassification bias, and could possibly influence the study results [39].

It is still unclear whether the number of stained teeth or the amount of staining present could predict the dental caries experience of the child. Among the twelve studies included in this review, (Chen, 2014) [15] was the only study that recorded the number of stained teeth; however, no attempt was made to correlate this with dental caries parameters.

Caries protective factors such as good oral hygiene, and infrequent use of nursing bottles was reported to be correlated with the number of stained teeth by Chen et al. (2014) [15], while Tripodi et al. (2016) [32] revealed that children who had BS reported higher fluoride intake than controls. These findings could imply that a non-cariogenic environment, might cause BS-forming organisms to flourish either by inhibiting the growth of cariogenic species or that the less acidic pH is more favorable for BS-formation [15]^.^ In other words, BS might not be the reason for the reduced dental caries in affected children, instead, its formation could merely be a secondary result of the caries-suppressing environment, as suggested by Slots’ (1974) [6] second hypothesis.

Garcia Martin et al. (2013) [12] did not find a statistical significance between BS and the level of oral hygiene but found that children with BS had significantly higher oral hygiene habits like toothbrushing with fluoride toothpaste, and using a fluoride mouth rinse. Conversely, three studies [22, 25, 31] did not find an association between toothbrushing frequency and BS, but one of them suggested that plaque removal efficiency might be a better predictor of BS formation than brushing frequency [22]. Sharaf et al. (2017) found no significant difference between groups in the scores of Simplified Oral Hygiene Index, frequency of toothbrushing, or age at which brushing started.

Tripodi et al. (2016) [32] reported that children with BS had a much higher consumption of sweets and soft drinks than the control group, but still experienced less dental caries. França-Pinto et al. (2012) [26] found no difference in sweets or sugary drinks consumption, while Garcia Martin et al. (2013) [12] reported that children with BS consumed less sugary drinks. Regarding other dietary habits, Garcia Martin et al. (2013) [12] stated that children with BS consumed more legumes, dairy products, bread, and eggs, but ate less fresh fruits, vegetables, and natural juices [12]. Sharaf et al. (2017) [31] also reported a significantly higher intake of dairy products, specifically white cheese in children with BS. As for pigmented foods, Chen et al. (2014) [15] reported that the BS group had a significantly higher consumption of soy sauce. França-Pinto et al. (2012) [26] reported that tap water is a risk factor for BS formation, while Sharaf et al. (2017) [31] found no association between BS and the source of drinking water. All of these dissonant findings leave much uncertainty regarding the relationship between diet and BS formation requiring further investigation.

Medication intake and systemic disease history are also factors that could affect both dental caries and black stain development. Garcia Martin et al. (2013) [12] reported that BS presence had a statistically significant relationship with the consumption of iron supplements by the child or the pregnant mother. Sharaf et al. (2017) [31] agreed that the consumption of iron supplements during pregnancy can significantly increase the risk of the child developing BS, but found no similar association with children’s consumption of iron or calcium supplements. Likewise, Chen et al. (2014) [15] reported that the intake of ferrous medicines by the child did not significantly affect the chance of BS formation or the number of stained teeth.

The medical history of both the child and the mother was deemed irrelevant by Sharaf et al. (2017) [31]. Elelmi et al. (2020) [25] reported a significant association between BS and history of chronic diseases or hospitalization (due to fever, asthma or pneumonia or others), while Chen et al. (2014) [15] also found a significant association between BS presence and history of pneumonia. The relation between BS and pneumonia could be worth further investigation since another microbiological study [40] suggested that *Enterobacter spp*—which is associated with some pneumonial infections—is significantly higher in children with BS.

Overall, there is a lack of consensus between the studies regarding the risk factors and the microbial etiology of BS. These findings could be attributed to the differences in age cohorts, dietary habits in different countries, or sampling techniques. Nevertheless, the association between BS and lower caries parameters seems to be sufficiently consistent in the available literature, regardless of our limited understanding of its underlying mechanisms.

## Limitations

One limitation of this study is that only studies reported in English language were assessed, thus valuable literature written in other languages could have been missed. The small number of studies did not allow exploring the effect of other confounding factors that could affect the relationship between BS and dental caries.

## Conclusions

The results of this study suggest that children with BS are less likely to develop dental caries, and have lower caries experience per tooth, and per surface than their peers. Many aspects regarding risk factors for BS formation, chemical composition of BS, oral microbiome of children with BS deposits remain unclear. It is also still unclear whether the severity of staining or number of stained teeth is associated with lower caries experience, or whether this caries protective effect of BS is limited to teeth/surfaces with BS or is generalized to the whole dentition. However, it is also still questionable whether children with BS are actually protected from dental caries, or it is that children at low risk of dental caries are just more likely to develop BS because their oral microbiome favors BS-forming organisms.

## Supplementary Information

**Additional file 1.**

## Data Availability

Supplementary data is available.

## Abbreviations

BS: Black stain
CI: Confidence interval
dmfs: decayed, missing, and filled surfaces
dmft: decayed, missing, and filled teeth
ECC: Early childhood caries
EULC: Egyptian Universities Libraries Consortium
GS: Google Scholar
IQR: Inter-quartile range
MD: Mean difference
OG: OpenGrey
OR: Odds ratio
PECO: Population, exposure, comparator, outcomes
PM: PubMed
SD: Standard deviation
SE: Standard error
WOS: Web of Science
