# Supplementary material for: The association between black stain and lower risk of dental caries in children: a systematic review and meta-analysis
Source: J Egypt Public Health Assoc. 2022 Jul 30;97:13. doi: 10.1186/s42506-022-00107-3 (PMC9338195; doi:10.1186/s42506-022-00107-3)
Supplement: Supplementary file 1 — Additional file 1. [file 42506_2022_107_MOESM1_ESM.docx]

S1:

1. PubMed search strategy

| #1 | “dental caries”[MeSH] |
| --- | --- |
| #2 | caries |
| #3 | “carious lesions” |
| #4 | “carious lesion” |
| #5 | “carious teeth” |
| #6 | “dental decay” |
| #7 | “tooth decay” |
| #8 | “decayed teeth” |
| #9 | cavities |
| #10 | dmfs |
| #11 | dmft |
| #12 | defs |
| #13 | deft |
| #14 | dft |
| #15 | dfs |
| #16 | #1 OR #2 OR #3 OR #4 OR #5 OR #6 OR #7 OR #8 OR #9 OR #10 OR #11 OR #12 OR #13 OR #14 OR #15 |
| #17 | “tooth discoloration” |
| #18 | “tooth discolorations” |
| #19 | “black stain” |
| #20 | “black stains” |
| #21 | “black pigmentation” |
| #22 | “black pigmentations” |
| #23 | “black discoloration” |
| #24 | “black discolorations” |
| #25 | “black tartar” |
| #26 | #17 OR #18 OR #19 OR #20 OR #21 OR #22 OR #23 OR #24 OR #25 |
| #27 | "child"[MeSH] |
| #28 | child |
| #29 | children |
| #30 | pediatr* |
| #31 | paediatr* |
| #32 | student* |
| #33 | pupil* |
| #34 | preschool* |
| #35 | “primary dentition” |
| #36 | “primary teeth” |
| #37 | “deciduous dentition” |
| #38 | “deciduous teeth”) |
| #39 | #27 OR #28 OR #29 OR #30 OR #31 OR #32 OR #33 OR #34 OR #35 OR #36 OR #37 OR #38 |
| #40 | #16 AND #26 AND #39 |

1. Web of Science search strategy

| #1 | TS= caries |
| --- | --- |
| #2 | TS= carious |
| #3 | TS= lesion* |
| #4 | #2 NEAR/3 #3 |
| #5 | TS= carious |
| #6 | TS= teeth |
| #7 | #5 NEAR/3 #6 |
| #8 | TS= dental |
| #9 | TS= decay |
| #10 | #7 NEAR/3 #8 |
| #11 | TS= tooth |
| #12 | TS= decay |
| #13 | #11 NEAR/3 #12 |
| #14 | TS= decayed |
| #15 | TS= teeth |
| #16 | #14 NEAR/3 #15 |
| #17 | TS= cavities |
| #18 | TS=“oral health outcomes” |
| #19 | TS=“oral health status” |
| #20 | TS=“dental outcomes” |
| #21 | TS=“dental health” |
| #22 | TS=dmfs |
| #23 | TS=dmft |
| #24 | TS=defs |
| #25 | TS=deft |
| #26 | TS=dft |
| #27 | TS=dfs |
| #28 | #1 OR #4 OR #7 OR #10 OR #13 OR #16 OR #17 OR #18 OR #19 OR #20 OR #21 OR #22 OR #23 OR #24 OR #25 OR #26 OR #27 |
| #29 | TS=“tooth discoloration” |
| #30 | TS=“tooth discolorations” |
| #31 | TS=“black stain” |
| #32 | TS=“black stains” |
| #33 | TS=“black pigmentation” |
| #34 | TS=“black pigmentations” |
| #35 | TS=“black discoloration” |
| #36 | TS=“black discolorations” |
| #37 | TS=“black tartar” |
| #38 | #29 OR #30 OR #31 OR #32 OR #33 OR #34 OR #35 OR #36 OR #37 |
| #39 | TS=child* |
| #40 | TS=pediatr* |
| #41 | TS=paediatr* |
| #42 | TS=pupil* |
| #43 | TS=student* |
| #44 | TS=preschool* |
| #45 | TS= “primary dentition” |
| #46 | TS= “primary teeth” |
| #47 | TS=“deciduous dentition” |
| #48 | TS=“deciduous teeth” |
| #49 | #39 OR #40 OR #41 OR #42 OR #43 OR #44 OR #45 OR #46 OR #47 OR #48 |
| #50 | #28 AND #38 AND #49 |

1. Scopus search strategy

| #1 | caries |
| --- | --- |
| #2 | “carious lesions” |
| #3 | “carious lesion” |
| #4 | “carious teeth” |
| #5 | “dental decay” |
| #6 | “tooth decay” |
| #7 | “decayed teeth” |
| #8 | cavities |
| #9 | dmfs |
| #10 | dmft |
| #11 | defs |
| #12 | deft |
| #13 | dft |
| #14 | dfs |
| #15 | #1 OR #2 OR #3 OR #4 OR #5 OR #6 OR #7 OR #8 OR #9 OR #10 OR #11 OR #12 OR #13 OR #14 |
| #16 | “tooth discoloration” |
| #17 | “tooth discolorations” |
| #18 | “black stain” |
| #19 | “black stains” |
| #20 | “black pigmentation” |
| #21 | “black pigmentations” |
| #22 | “black discoloration” |
| #23 | “black discolorations” |
| #24 | “black tartar” |
| #25 | #16 OR #17 OR #18 OR #19 OR #20 OR #21 OR #22 OR #23 OR #24 |
| #26 | child |
| #27 | children |
| #28 | pediatr* |
| #29 | paediatr* |
| #30 | student* |
| #31 | pupil* |
| #32 | preschool* |
| #33 | “primary dentition” |
| #34 | “primary teeth” |
| #35 | “deciduous dentition” |
| #36 | “deciduous teeth”) |
| #37 | #26 OR #27 OR #28 OR #29 OR #30 OR #31 OR #32 OR #33 OR #34 OR #35 OR #36 |
| #38 | #15 AND #25 AND #37 |

S2: Summary of included and excluded studies

|  | Author/date | Result | Reason for exclusion |
| --- | --- | --- | --- |
| 1 | {Akyuz, 2015 }^1^ | **✓** |  |
| 2 | {Antunes, 2006 }^2^ | ✘ | Does not assess primary dentition |
| 3 | {Aysun, 2012 }^3^ | **✓** |  |
| 4 | {Bircher, 2008 }^4^ | ✘ | dmf/DMF are not reported separately |
| 5 | {Boka, 2013 }^5^ | **✓** |  |
| 6 | {Chen, 2014 }^6^ | **✓** |  |
| 7 | {Dobbiani, 2018 }^7^ | ✘ | Did not assess the presence of black stain |
| 8 | {Elelmi, 2020} | **✓** |  |
| 9 | {França-Pinto, 2012 }^8^ | **✓** |  |
| 10 | {Frencken, 1988 }^9^ | ✘ | Does not measure the association between black stain and caries |
| 11 | {Garcia Martin, 2013 }^10^ | **✓** |  |
| 12 | {Gasparetto, 2003 }^11^ | ✘ | Does not assess primary dentition |
| 13 | {Heinrich-Weltzien, 2009 }^12^ | ✘ | Does not assess primary dentition |
| 14 | {Heinrich‐Weltzien, 2014 }^13^ | **✓** |  |
| 15 | {Hwang, 2020}^14^ | **✓** |  |
| 16 | {Koch, 2001 }^15^ | **✓** |  |
| 17 | {Lopez Martinez, 2016}^16^ | ✘ | dmf/DMF are not reported separately |
| 18 | {Mathews, 2020} | ✘ | dmf/DMF are not reported separately |
| 19 | {Muthu, 2019 }^17^ | **✓** |  |
| 20 | {Mutsaddi, 2018 }^18^ | **✓** |  |
| 21 | {Ness, 1977 }^19^ | ✘ | Does not assess the presence of black stain |
| 22 | {Núñez, 2015 }^20^ | ✘ | Article in Spanish |
| 23 | {Panagidis, 2012 }^21^ | ✘ | Does not assess primary dentition |
| 24 | {Hassan, 2008 }^22^ | ✘ | Does not assess the presence of black stain |
| 25 | {Reid, 1977 }^23^ | ✘ | Does not assess primary dentition |
| 26 | {Sari, 2011 }^24^ | ✘ | Does not assess primary dentition |
| 27 | {Sharaf, 2017 }^25^ | **✓** |  |
| 28 | {Tirth, 2009 }^26^ | ✘ | Does not assess primary dentition |
| 29 | {Tripodi, 2016 }^27^ | **✓** |  |
| 30 | {Velásquez Sáez, 2017 }^28^ | ✘ | Article in Spanish |
| 31 | {Wong, 2001 }^29^ | ✘ | Does not assess the presence of black stain |

1. Akyuz S, Garan A, Kaya M. Prevalence of black stain and dental caries in children attending a university pediatric dentistry clinic in Istanbul. *J Marmara Univ Inst Heal Sci*. 2015;5(2):109-114. doi:10.5455/musbed.20150414123805

2. Antunes JLF, Peres MA, Mello TRDC, Waldman EA. Multilevel assessment of determinants of dental caries experience in Brazil. *Community Dent Oral Epidemiol*. 2006;34(2):146-152. doi:10.1111/j.1600-0528.2006.00274.x

3. Garan A, Akyüz S, Oztürk LK, Yarat A. Salivary parameters and caries indices in children with black tooth stains. *J Clin Pediatr Dent*. 2012;36(3):285-288. doi:10.17796/jcpd.36.3.21466m672t723713

4. Bircher M, Luca A, Sutich E. Black Stain and Caries in Deciduous and Mixed Dentition. 2008.

5. Boka V, Trikaliotis A, Kotsanos N, Karagiannis V. Dental caries and oral health-related factors in a sample of Greek preschool children. *Eur Arch Paediatr Dent*. 2013;14(6):363-368. doi:10.1007/s40368-013-0097-5

6. Chen X, Zhan J-Y, Lu H-X, et al. Factors associated with black tooth stain in Chinese preschool children. *Clin Oral Investig*. 2014;18(9):2059-2066. doi:10.1007/s00784-013-1184-z

7. Dobbiani A, Berton F, Perinetti G, Costantinides F, DI Lenarda R. Prevalence of dental caries among schoolchildren from North-Eastern Italian population. *Minerva Stomatol*. 2018;67(2):49-54. doi:10.23736/S0026-4970.17.04041-9

8. França-Pinto CC, Cenci MS, Correa MB, et al. Association between Black Stains and Dental Caries in Primary Teeth: Findings from a Brazilian Population-Based Birth Cohort. *Caries Res*. 2012;46(2):170-176. doi:10.1159/000337280

9. Frencken JEFM. Dental caries in a Tanzanian child population: a mixed-longitudinal approach, PhD dissertation, Katholieke Universiteit Nijmegen. 1988.

10. Garcia Martin JM, Gonzalez Garcia M, Seoane Leston J, Llorente Pendas S, Diaz Martin JJ, Garcia-Pola MJ. Prevalence of black stain and associated risk factors in preschool Spanish children. *Pediatr Int*. 2013;55(3):355-359. doi:10.1111/ped.12066

11. Gasparetto A, Conrado CA, Maciel SM, Miyamoto EY, Chicarelli M, Zanata RL. Prevalence of black tooth stains and dental caries in Brazilian schoolchildren. *Braz Dent J*. 2003;14(3):157-161. doi:10.1590/S0103-64402003000300003

12. Heinrich-Weltzien R, Monse B, Helderman WVP. Black stain and dental caries in Filipino schoolchildren. *Community Dent Oral Epidemiol*. 2009;37(2):182-187. doi:10.1111/j.1600-0528.2008.00458.x

13. Heinrich-Weltzien R, Bartsch B, Eick S. Dental Caries and Microbiota in Children with Black Stain and Non-discoloured Dental Plaque. *Caries Res*. 2014;48(2):118-125. doi:10.1159/000353469

14. Hwang JY, Lee H-S, Choi J, Nam OH, Kim MS, Choi SC. The Oral Microbiome in Children with Black Stained Tooth. *Appl Sci*. 2020;10(22):8054. doi:10.3390/app10228054

15. Koch MJ, Bove M, Schroff J, Perlea P, García-Godoy F, Staehle HJ. Black stain and dental caries in schoolchildren in Potenza, Italy. *ASDC J Dent Child*. 2001;68(5-6):353-355, 302.

16. López Martínez TM, Goettems ML, Azevedo MS, Correa MB, Demarco FF, Romano AR. Black stains and dental caries in Brazilian schoolchildren: a cross-sectional study. *Braz Oral Res*. 2016;30(1):e110. doi:10.1590/1807-3107BOR-2016.vol30.0110

17. Muthu MS, Saikia A, Henry JA, Balamurugan A. Surface-specific Correlation Between Extrinsic Stains and Early Childhood Caries. *Oral Health Prev Dent*. 2019;17(3):277-282. doi:10.3290/j.ohpd.a42205

18. Mutsaddi S, Kotrashetti VS, Nayak R, Pattanshetty S, Hosmani J V., Babji D. Association of dental caries in children with black stain and non-discolored dental plaque: A microbiological study. Nagaraj T, ed. *J Adv Clin Res Insights*. 2018;5(3):59-64. doi:10.15713/ins.jcri.212

19. Ness L, Rosekrans DL, Welford JF. An epidemiologic study of factors affecting extrinsic staining of teeth in an English population. *Community Dent Oral Epidemiol*. 1977;5(1):55-60. doi:10.1111/j.1600-0528.1977.tb01617.x

20. Núñez NCG, Vergara SSJS, Mondaca ÁNP, Villagrán PBM, Castro NV. Frequency of dental stains in schoolchildren of San Juan de la Costa, Chile, 2012. *Rev Cubana Estomatol*. 2015;52n(1):2-10.

21. Panagidis D, Schulte AG. Caries prevalence in 12-year-old Cypriot children. *Community Dent Health*. 2012. doi:10.1922/CDH_2774Panagidis05

22. Hassan ZS, Qasim AA. Oral hygiene condition among five years old Kindergarten Children in relation to level of parent education in Baghdad city Iraq. *J Fac Med*. 2008;50(4):440-444.

23. Reid JS, Beeley JA. Biochemical Studies on the Composition of Gingival Debris from Children with Black Extrinsic Tooth Stain. *Caries Res*. 1976;10(5):363-369. doi:10.1159/000260217

24. Sari O, Ertan T, Ertugrul C, Sahinkaya E. Comperative Evaluation of Diagnostic Profile, Age, Sex and Educational Background of Patients to Have Referred to Dental Clinic for Tooth and Gingival Diseases. *TAF Prev Med Bull*. 2011;10(5):565-572. doi:10.5455/pmb.20110220032205

25. Sharaf R, Kabil N, Abou El Fadl R. Assessment of the prevalence of extrinsic tooth stains and identification of potential risk factors in a group of Egyptian children. Master’s degree thesis, 2017. Pediatric Dentistry and Dental Public Health Department, Faculty of Dentistry, Ain Shams Uni.

26. Tirth A, Srivastava B, Nagarajappa R, Ravishankar T. An Investigation into Black Tooth Stain Among School Children in Chakkar Ka Milak of Moradabad City, India. *J Oral Heal Community Dent*. 2009;3(2):34-37. doi:10.5005/johcd-3-2-34

27. Tripodi D, Martinelli D, Pasini M, Giuca MR, D’Ercole S. Black Stains: a microbiological analysis and a view on familiarity and susceptibility to tooth decay of patients in childhood. *Eur J Paediatr Dent*. 2016;17(4):261-266.

28. Velásquez Sáez C, Salinas Villanueva I, Godoy Martínez P, Muñoz Martínez H, Barría Pailaquilén RM. Counting of Streptococcus mutans in saliva of 6 to 12 years old children with and without black stain. *Av Odontoestomatol*. 2017;33(2):77-85.

29. Wong MCM, Lo ECM, Schwarz E, Zhang HG. Oral Health Status and Oral Health Behaviors in Chinese Children. *J Dent Res*. 2001;80(5):1459-1465. doi:10.1177/00220345010800051501
